# Digital Health Technologies in Clinical Trials: An Ontology-Driven Analysis to Inform Digital Sustainability Policies

**DOI:** 10.1007/s43441-023-00560-y

**Published:** 2023-08-06

**Authors:** Spencer Phillips Hey, Maria Dellapina, Kristin Lindquist, Bert Hartog, Jason LaRoche

**Affiliations:** 1 Prism Analytic Technologies, 245 Main St., Cambridge, MA 02142 USA; 2 Janssen-Cilag B.V., 4837 DS Breda, The Netherlands; 3 Janssen Research & Development, LLC, Raritan, NJ 08869 USA

**Keywords:** Digital health, Clinical trials, Decentralized trials, Environmental sustainability, Research policy

## Abstract

**Background:**

Digital health technologies (DHTs) can facilitate the execution of de-centralized trials that can offer opportunities to reduce the burden on participants, collect outcome data in a real-world setting, and potentially make trial populations more diverse and inclusive. However, DHTs can also be a significant source of electronic waste (e-waste). In recognition of the potential health and environmental impact from DHT use in trials, private and public institutions have recently launched initiatives to help measure and manage this e-waste. But in order to develop sound e-waste management policies, it will be necessary to first estimate the current volume of e-waste that results from the use of DHTs in trials.

**Materials and Methods:**

A Web Ontology Language (OWL)-compliant ontology of DHTs was created using a list of 500 DHT device names derived from a mixture of public and private sources. The U.S. clinical trials registry, ClinicalTrials.gov, was then queried to identify and classify trials using any of the devices in the ontology. The ClinicalTrials.gov records from this search were then analyzed to characterize the volume and properties of trials using DHTs, as well as estimating the total volume of individual DHT units that have been provisioned (or are planned to be provisioned) for clinical research.

**Results:**

Our ontology-driven search identified 2326 unique clinical trials with a reported “actual” enrollment of 200,947 participants and a “planned” enrollment of an additional 4,094,748 participants. The most-used class of DHTs in our ontology was “wearables,” (1852 trials), largely driven by the use of smart watches and other wrist-worn sensors (estimated to involve 149,391 provisioned devices). The most-used subtype of DHTs in trials was “subcutaneous” devices (367 trials), driven by the prevalent use and testing of glucose monitors (estimated to involve 17,666 provisioned devices).

**Conclusion:**

Thousands of trials, involving hundreds of thousands of devices, have already been completed, and many more trials (potentially involving millions more devices) are planned. Despite the great opportunities that are afforded by DHTs to the clinical trial enterprise, if the industry lacks the ability to track DHT use with sufficient resolution, the result is likely to be a great deal of e-waste. A new ontology of DHTs, combined with rigorous data science methods like those described in this paper, can be used to provide better information across the industry, and in turn, help create a more sustainable and equitable clinical trials enterprise.

**Supplementary Information:**

The online version contains supplementary material available at 10.1007/s43441-023-00560-y.

## Introduction

The proliferation of digital health technologies (DHTs) represents a double-edged sword for the clinical trial enterprise. On the one hand, DHTs can facilitate the execution of de-centralized trials that can offer opportunities to reduce the burden on participants, collect outcome data in a real-world setting, and potentially make trial populations more diverse and inclusive [1–3]. For example, distributing smart watches to participants to collect the bulk of outcomes data can eliminate the need for patients to travel to health centers in order to participate in research [1, 2]. This opens up possibilities for conducting research in remote or otherwise less resourced areas, thereby creating opportunities to address some long-neglected health disparities [1, 2]. Where DHTs have been sufficiently validated as health-outcome collection instruments, they can also help make clinical trials more informative [2, 3]. For example, validated actigraphy devices can collect longitudinal data to better understand participants’ regular activity or sleep patterns over a longer period as compared to the more discrete timeframe measures from surveys [4] or on-site sleep monitoring experiments [5].

On the other hand, DHTs can be a significant source of electronic waste (e-waste), since these devices contain a mixture of plastics, critical or rare metals, and toxic chemicals that are harmful to the environment [6–8]. A prime example of the ‘make-take-dispose’ economic model [9], DHTs are typically designed for optimal safety and performance, but not eventual reuse and recycling [10]. For example, device batteries are often difficult or impossible to remove, which can present fire and explosion risks during mechanical processing. Some DHTs may also pose unique biological (infectious disease) or chemical (trace drug product) risks which, for their current single-use design, makes them unsuitable for any material recovery activity. [10]

In recognition of the potential health and environmental impact from DHT use in trials, private and public institutions have recently launched initiatives to help measure and manage this e-waste [6–8]. One approach is transforming the design of DHTs such that they can be easily maintained, reused, and refurbished, while still maintaining high standards of safety and performance. Within this ‘circular’ economic model, recycling and disposal are considered last resorts as opposed to the status quo [9]. However, in order to develop sound e-waste management policies and work towards a truly circular economy, it will be necessary to first estimate the current volume of e-waste that results from the use of DHTs in trials.

To our knowledge, only one prior study has attempted to quantify the number of trials that used connected DHTs, analyzing ClinicalTrials.gov data up to the end of 2018 [11]. According to this study, approximately 4% of all trial registered with start dates in 2017 and 2018 on ClinicalTrials.gov included some kind of DHT. However, this study used a broad, keyword-based, search strategy and ad hoc methodology that, while likely to be sensitive and sufficient for the purpose of a one-off publication, is inherently non-specific and insufficient for supporting sound evidence-based policies for managing e-waste. Sound policies for this rapidly evolving domain demand a repeatable and scalable approach that permits decision-makers to monitor and drill deeper into the evolving state of DHT use at any time.

The purpose of the present study is thus to develop a novel data science approach that is capable of generating an estimate for the volume of use for specific DHTs in a repeatable, scalable, and transparent fashion, thereby giving decision- and policy-makers ongoing insight into the use of DHTs in trials and its potential environmental impact.

## Materials and Methods

### Data Sources

The primary source of clinical trial data for this study was ClinicalTrials.gov. However, since ClinicalTrials.gov does not contain-specific fields for DHTs, we also used clinical trial datasets provided by Janssen to estimate the coverage of our search and analysis algorithms.

### DHT Ontology

We built a Web Ontology Language (OWL)-compliant ontology of DHTs using a list of device names derived from a mixture of public and private sources (e.g., Janssen’s internal DHT use data, Human First Atlas), as well as our own pilot searches of ClinicalTrials.gov. The OWL standard is a semantic markup language for structuring data that was developed to facilitate the rigorous scientific analysis of data and exchange of knowledge over the internet [12]. At the time of writing, this ontology includes 39 entity classes and 500 individual DHTs. The ontology is available to view and download on BioPortal, a repository for biomedical ontologies (https://bioportal.bioontology.org/ontologies/ODHT) hosted by Stanford University’s Center for Biomedical Informatics Research.

For each of the 500 devices in our ontology, we searched the internet for device specifications or a user manual for the information necessary to populate its properties—i.e., classifying each individual device by its type (e.g., wearable smartwatch), as well as associating it with a specific manufacturer and its measurement components (e.g., accelerometer, thermometer). Table S1 contains the tabular representation of all the data from our ontology.

### Search Strategy

To estimate the number of trials that involved DHTs, we wrote a Python (python.org) script that looped through all of the DHTs in our ontology, querying ClinicalTrials.gov for any records that included the device’s model name and manufacturer as keywords. There were no other restrictions on this search (e.g., we did not limit the search by date, study type, phase, or geography).

For DHTs that have “generic” model and manufacturer names (e.g., Lumen), we constructed a more targeted search string that included additional descriptive terms (e.g., “metabolism device”) to reduce the type-1 errors. For cases where different models of device have very similar names (e.g., “FitBit Charge” and “FitBit Charge 2”), a Boolean search string was constructed that would attempt to pick out only the device model of interest (e.g., “FitBit Charge” NOT “Charge 2”). Table S1 includes the exact ClinicalTrials.gov search string we used for every device in the ontology (hyperlinked to allow for inspection of every result set), as well as the number of records returned and the NCT identification numbers for each record.

Since trials may involve a DHT without mentioning a specific DHT by name and manufacturer, a broader, keyword-based search strategy was also applied [11], which included many more general terms and references to DHTs (e.g., “smartwatch”, “mhealth”). While this approach is far more inclusive, it cannot reliably associate particular devices with particular trials without laborious manual review of every record, and thus, this method cannot be repeatedly performed at scale. Therefore, this broader search was only used for the purposes of a sensitivity analysis, representing a possible upper bound on the number of trials that involved DHTs. Table S2 includes the complete results of this keyword-driven approach. This analysis is publicly available on the Prism meta-research platform (https://app.prism.bio/p/323/dht-keyword-analysis).

### Analysis

To estimate the total number of trials that involve DHTs, we wrote another Python script that generates the set of unique ClinicalTrials.gov records from our ontology-driven search results. This script essentially “pivots” our DHT search results into a new table, where every unique trial is a row that includes the trial’s start date, lead sponsor, type of sponsor (e.g., industry, government), purpose, phase, actual enrollment (or planned enrollment, if the actual enrollment is not yet reported in the record), the maximum timeframe among the reported outcomes (“max timeframe”), trial status, therapeutic area, and countries of recruitment. For each trial record, we also derived its therapeutic area from any Medical Subject Heading (MeSH) terms included in the record. We ran this final data extraction on August 23, 2022 using Prism’s clinical trials application programming interface (which contains a copy of ClinicalTrials.gov with data transformations and classifications to make the data more readable and useful).

To calculate the total number of devices, we generally assumed 1 device per trial participant, except in the following cases:

- For contact lenses and ingestibles, we assumed 1 device per participant for every day of the max timeframe.
- For sensor patches, we assumed 1 device per participant every 7 days of the max timeframe.
- For blister packs, we assumed 1 device per participant every 30 days of the max timeframe.
- For syringes, we assumed 1 device per participant for every 90 days of the max timeframe.

These assumptions were based upon detailed trial designs with which the study authors have first-hand experience. We assumed that the operational aspects of these trials were more broadly applicable.

## Results

Our ontology-driven search for 500 DHTs identified 2326 unique clinical trials (as of August 23, 2022) with a reported “actual” enrollment of 200,947 participants and a “planned” enrollment of an additional 4,094,748 participants. The distributions of DHTs across the device classes and subtypes in our ontology, trial prevalence, and participant counts are described in Table 1.

**Table 1.** Distribution of DHTs in the Ontology with Estimated Number of Devices (D).

	Devices	Trials	D Actual	D Planned
Total	500	2326	289,222	5,708,221
Wearable	359	1852	195,642	1,833,013
Sensor box	180	1310	116,327	212,228
Watch	142	450	33,064	1,079,781
Sensor patch	22	65	26,744	311,802
Transmitter	13	338	16,467	27,391
Clothing clip	12	59	6060	1166
Smart clothing	8	25	547	1337
Other	1	0	0	0
Glasses	1	5	208	100
Contact lens	1	37	23,342	235,247
Non-wearable	115	341	58,305	140,892
Handheld	51	52	28,596	18,783
Blood test kit	27	67	13,838	2715
Scale	14	67	4141	8676
Arm cuff	13	23	7039	1517
Receiver	11	178	11,269	10,005
Remote monitor	5	8	1217	100,132
Clip	5	6	36	476
Speaker	1	0	0	0
Other	19	385	33,417	53,345
Subcutaneous	16	367	17,666	29,386
Ingestible	3	18	15,751	23,959
Packaging	18	30	3,647	2580
Blister	5	2	1248	800
Cap	4	7	240	560
Inhaler	3	17	1881	1220
Dispenser	3	3	178	0
Syringe	2	1	100	0
Vial	1	0	0	0
Lifestyle	11	250	23,657	3,715,619
Phone	4	51	15,911	3,708,905
Game console	4	157	5773	4290
Tablet	1	30	1578	1570
Portable media player	1	6	199	286
Home assistant	1	12	196	568

The values within each category do not sum to the total, because a DHT can involve both a wearable and a non-wearable element. Similarly, a single trial can involve multiple devices with multiple classifications and subtypes. D is the estimated number of devices, either “actual” (calculated from actual trial participants) or “planned” (calculated from anticipated or target trial enrollment)

The largest class of DHTs in our ontology was “wearables,” (359, 71%) which included sensor boxes (180), smart watches (142), and sensor patches (23) as the most numerous subtypes. “Non-wearable” DHTs (115, 23%) were the next most numerous class of devices, which included handheld devices (51), blood test kits (27), smart scales (14), and arm cuffs (13) as subtypes. Table S3 shows the definitions used for each class and subtype in our ontology, as well as exemplar devices for each subtype.

Wearable DHTs were found in 1852 trial records, with sensor boxes (1310 trials) being the most frequently reported subtype. “Other” DHTs—which included ingestible and subcutaneously implanted devices—were the next most numerous class, found in 385 trials. The “subcutaneous” subtype accounted for nearly all of these instances (367 trials, 95%), driven by the prevalent use of continuous glucose monitors. The receiver device subtype, which accounted for the largest share of the non-wearable DHTs (178 out of 341 trials, 52%), was similarly driven by its use as a component of continuous glucose monitors.

Despite a relatively small number of “Lifestyle” devices in our ontology (11 DHTs), this class was found in a relatively large number of trial records (250 trials). The game console subtype from this class was the most frequently identified (157 trials), driven by the Nintendo Wii and Wii Balance Board, which were identified in a combined 94 trial records.

Using “actual” trial participants to estimate the number of individual DHTs thus far provisioned in trials, the wearable class of devices was found to be the most numerous (195,642), with sensor boxes being the most frequent device subtype (116,327). Non-wearable devices were the second most numerous (58,305), with handheld DHTs being the most frequent subtype (28,596). The Lifestyle class devices were the next most numerous (23,657), but rather than game consoles (which were involved in the most trials) driving these numbers, it was phones that were the most frequent subtype (15,911). However, using “planned” trial participants as an estimate for the number of individual DHTs that may be provisioned for use in trials in the near-future, phones are by far the most numerous (3,708,905). Watches are the second most numerous (1,079,781); wearable sensor patches a distant third (311,802).

Table 2 describes the properties of the trials themselves. Most of the trials identified by our search were interventional studies (1885, 81%). The majority also described themselves as “phase not applicable” (2138, 92%), which suggests that the majority of trials using DHTs are either not testing products seeking regulatory approval or are evaluating interventions (e.g., behavioral interventions) for which the phased model of development is not relevant. Just under half of the trial records had a status of “Completed” (1136, 49%), followed by the statuses of “Recruiting” (523, 22%) and “Unknown” (202, 9%).

**Table 2.** Properties of Clinical Trials Identified as Including DHTs.

	Trials	N Actual	N Planned
Total	2326	200,947	4,094,748
Study type			
Interventional	1885	149,342	2,396,065
Observational	441	51,605	1,698,683
Phase			
Not applicable	2138	189,213	4,069,437
Phase 2	61	2409	2219
Phase 4	58	3900	2069
Phase 1	39	1006	966
Phase 3	30	4419	20,057
Status			
Completed	1142	163,093	18,447
Recruiting	523	0	3,898,436
Unknown	202	9145	20,838
Not yet recruiting	179	0	31,124
Active not recruiting	125	23,387	16,172
Terminated	59	5302	106
Withdrawn	46	0	0
Enrolling by invitation	40	0	108,057
Suspended	10	20	1568
Sponsor type			
University	1174	110,724	2,182,904
Research hospital	415	33,803	1,039,850
Industry	351	26,988	828,521
Other	289	26,342	34,672
Government	97	3090	8801
Disease area (top 10)			
Pathological conditions signs and symptoms	678	68,891	1,358,898
Nutritional and metabolic diseases	538	47,051	40,087
Nervous system diseases	452	15,089	285,028
Endocrine system diseases	378	28,956	21,871
Cardiovascular diseases	312	41,056	1,080,615
Immune system diseases	271	15,022	17,931
Mental disorders	214	6740	39,148
Respiratory tract diseases	178	14,642	115,318
Neoplasms	111	2967	7298
Physiological phenomena	97	8566	6454
Country of recruitment (top 10)			
United States	910	71,635	3,865,700
Canada	162	7368	7912
United Kingdom	134	26,675	9,313
Spain	103	10,130	10,261
France	102	3800	13,227
Germany	89	9054	11,362
Denmark	74	5457	6759
Brazil	59	2197	1212
Switzerland	57	4934	3549
Belgium	56	4132	4590

Universities were the largest lead sponsor group (1174 trials, 50%), and their trials also reported the largest numbers of actual (110,724) and planned participants (2,182,904). Research hospitals were the second most frequent sponsor type (407 trials, 17%; 33,803 actual participants; 1,038,850 planned participants), followed closely by industry (351 trials, 15%; 26,988 actual participants; 828,521 planned participants). This proportion is somewhat lower than the baseline balance between industry-sponsored studies (approximately 24%) and non-industry-sponsored studies.

Although only 39% of trials were recruiting in the United States (910 trials), these trials constituted the largest portion of participants, both actual (71,635, 36%) and planned (3,865,700, 94%). Canada was the country with the second most trials (162), followed by several countries in Western Europe (United Kingdom 134, Spain 103, France 102, Germany 89). However, the apparent emphasis on trials in the United States (and Canada) is to be expected given that the data source is the United States’ trial registry.

Pathological conditions signs and symptoms was the most studied therapeutic area (678, 29%), although this is not an especially informative area categorization, and the volume of trials classified as falling into this disease area is likely an artefact of the MeSH tree structure. However, the next most studied areas are more informative categories and include: nutritional and metabolic diseases (538 trials), nervous system diseases (452 trials), endocrine (378 trials), cardiovascular (312 trials), and immune system diseases (271 trials).

In terms of participants in these more meaningful categories, nutritional and metabolic diseases constituted the largest group of actual participants (47,051), followed closely by cardiovascular system diseases (41,056). Cardiovascular diseases were by far the largest in terms of planned participants (1,080,615); however, this number is almost entirely driven by a single observational trial that is planning to enroll a million participants in an evaluation of the Apple Watch’s ability to collect clinically meaningful electrocardiogram data (NCT05324566). Excluding this one cardiovascular disease trial, trials for nervous system (285,028) and respiratory tract (115,318) diseases have the greatest number of planned participants.

**Figure 1.**
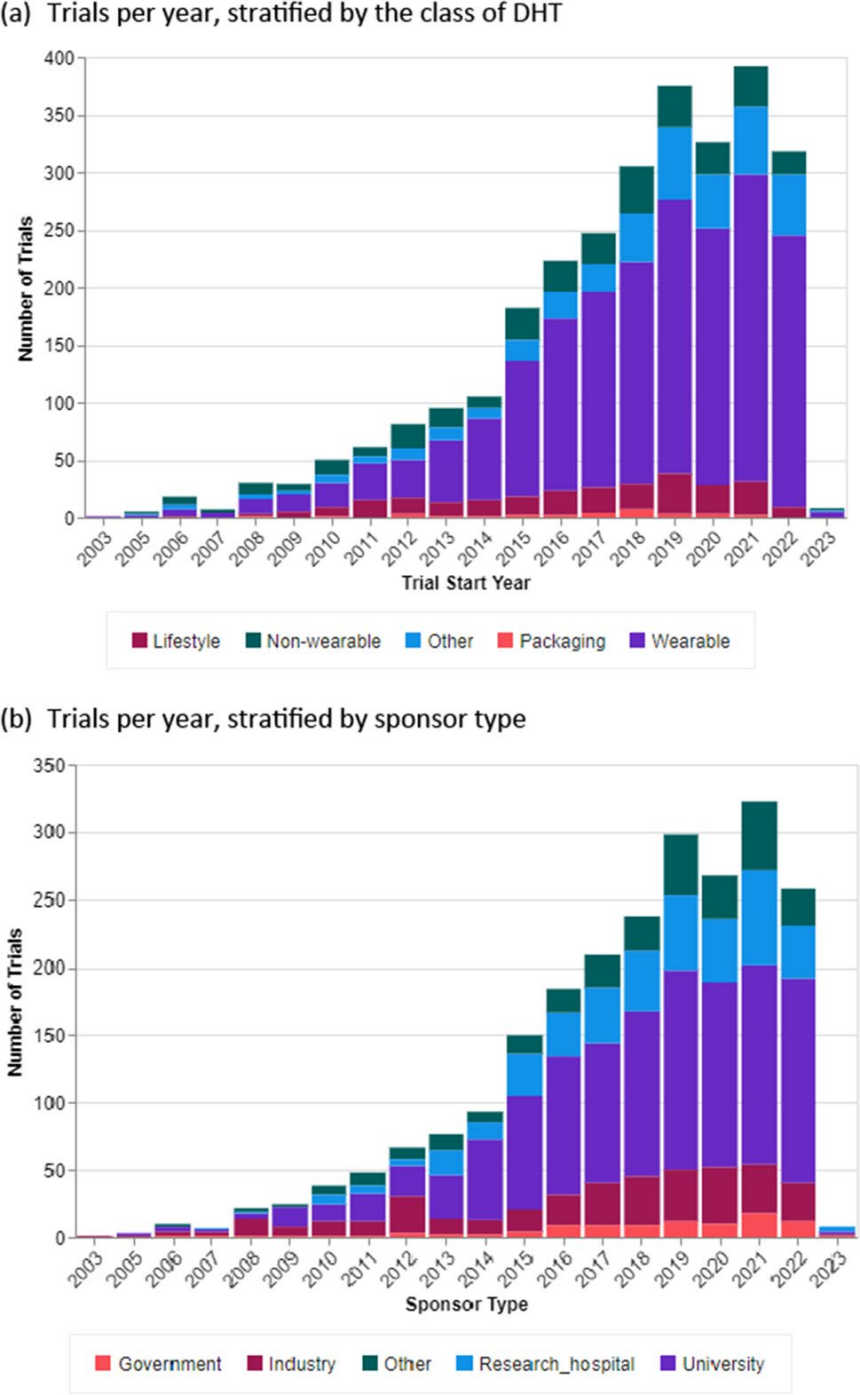
Volume of Clinical Trials Incorporating DHTs Over Time. **a** Trials Per Year, Stratified by the Class of DHT. **b** Trials Per Year, Stratified by Sponsor Type.

Figure 1 depicts the trends in trial volume over time, showing the number of trials initiated each year (using the trial record’s reported start date), stratified by DHT class (Fig. 1a) or sponsor type (Fig. 1b). University sponsors and wearable devices appear to be the consistent drivers of trial activity and DHT use over time. However, our results indicate a spike in activity in 2015, largely due to increased trial activity from research hospitals. Industry activity also spiked around this time (2016), where it jumped from a previous average of less than 20 trials per year involving DHTs to averaging more than 30 trials per year.

By contrast, Fig. 1a shows recent (post 2020) downward trends in activity for lifestyle and non-wearable devices. In the case of lifestyle devices, this appears to be driven by game consoles—specifically, the Nintendo Wii and Microsoft Kinect. Research use of these devices appears to have blossomed two years after each device was released (2008 and 2010, respectively), but has tapered off sharply since 2020. In the case of non-wearable devices, the majority of research activity in this category was driven by “receiver” devices—which was, until recently, a common component in glucose monitors. However, with later models of glucose monitor DHTs, the data are often relayed to a smartphone, smartwatch, or computer and hence there is no need for a non-wearable receiver component.

**Figure 2.**
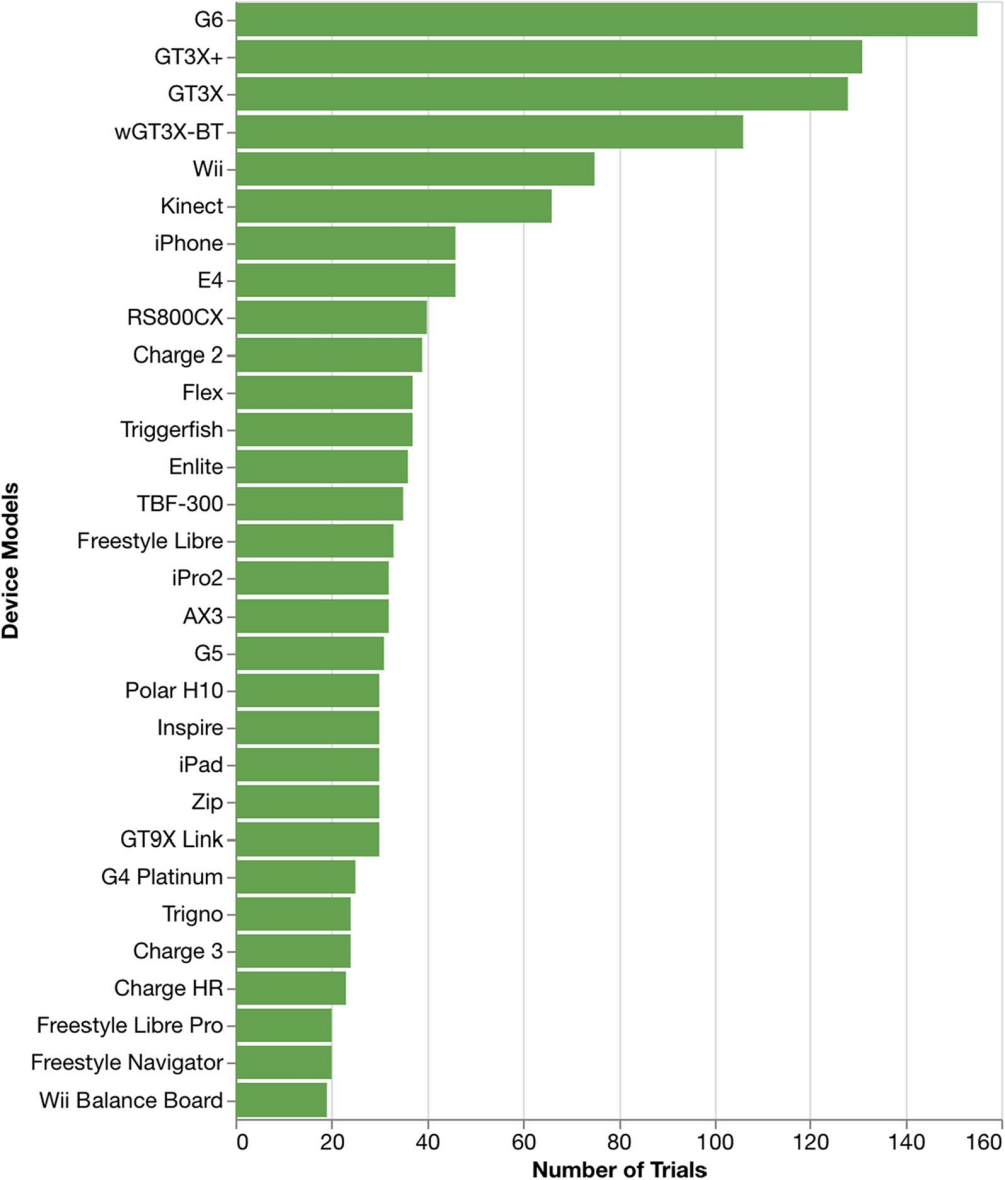
Number of Trials for the Top 30 Most-Used DHTs.

**Figure 3.**
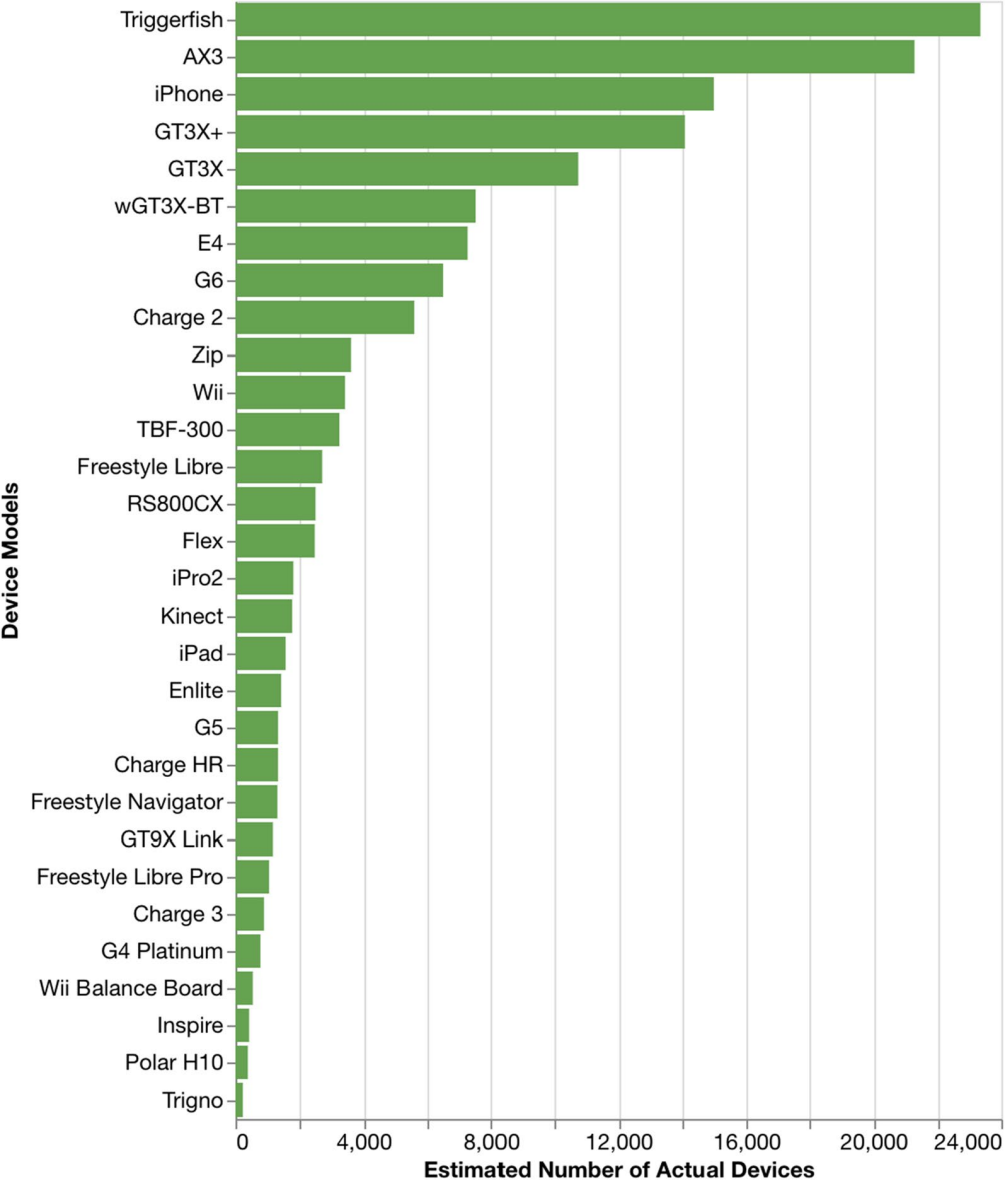
Estimated Number of Device Units for the Top 30 Most-Used DHTs.

Figure 2 shows the number of trials for the top 30 most-used DHTs. The Dexcom G6 continuous glucose monitor is the most-used DHT in our ontology (155 trials), followed by a suite of ActiGraph sensor box devices (GT3X + 131 trials; GT3X 128 trials; wGT3X-BT 106 trials), and the Wii video game console (75 trials). Figure 3 shows the estimated number of device units for these same 30 DHTs. We estimate that the Sensimed Triggerfish contact lens is the most provisioned device (23,342 units), followed by the Axivity AX3 wearable accelerometer (21,267 units), Apple iPhone (14,976 units), and then the suite of ActiGraph devices (GT3X + 14,094; GT3X 10,736; wGT3X-BT 7533). However, planned participant counts suggest that more than 3.7 million iPhones may be provisioned for use in trials, which is orders of magnitude greater than the next most “planned” device (ActiGraph wGT3X-BT at 18,044 units).

## Discussion

To inform the development of digital sustainability policies, we’ve demonstrated a scalable and reproducible method to generate a high-resolution (albeit limited coverage) picture of DHT use in clinical trials. After compiling a structured ontology of 500 DHTs, we found 2326 clinical trial records that reference these devices. These trials report 200,947 participants already enrolled—which we estimate corresponds to approximately 289,222 individual DHTs provisioned for use in trials.

However, over four million more participants are expected to be recruited, which if successful, would potentially represent millions more devices being provisioned for use in trials. While this is an exciting prospect and speaks to the growing importance of DHTs to the clinical trial enterprise, it also represents a large volume of trial-related e-waste, and therefore, underscores the importance of tracking this data to inform appropriate digital sustainability policies for the industry.

While we defer to environmental policy experts about whether the volume of e-waste represented in our estimates is “good” or “bad,” we do believe that our results can serve as a valuable benchmark for the research industry. For example, as our ontology grows to include a larger portion of devices, the analytic method we’ve employed can be easily repeated over time to rigorously track progress toward sustainability targets, for both individual institutions as well as the industry at large.

Indeed, since many stakeholders (across healthcare institutions, device manufactures, academia and pharmaceutical companies) have committed to net-zero emissions targets [13], the approach we’ve described here can contribute to sound, evidence-based policies that will enable the community to gain control over the global impact of emissions. For example, our method, if scaled, would support ongoing monitoring of actual device use, both at the device level (what devices are used, in what quantity, and total emissions) and for monitoring the environmental impact of specific locations (e.g., estimating the local emission levels for specific manufacturers).

Similarly, if DHT reporting standards improve (e.g., by adding dedicated fields to trial registries) it will be possible to use these same methods to provide a more accurate picture of not just the e-waste, but also the benefits that result from DHT use in trials. National regulatory agencies, for example, have shown an openness to digital endpoints in trials—but at present, due to gaps in reporting, it is difficult to know which trials have used DHTs to measure trial endpoints, much less which (if any) of those endpoints has been accepted by a regulator. Illuminating this picture of the DHT landscape for regulatory approval would thus seem to have global value—for industry, for regulators, and for academic researchers alike.

Much of the same can be said for so-called “bring-your-own-device” (BYOD) trials, which make use of research participants’ personal DHTs. Since BYOD approaches can have a negligible climate impact when the DHTs involved are not fully dedicated and are widely used for other purposes, a reporting element to trial registries that also allow sponsors to declare their studies as BYOD would be another valuable addition in support of sustainability policies.

The need for high-resolution evidence to inform sound policy is even more pressing when we consider that our estimates represent a lower-bound on the total volume of DHT use. Through the adoption of circular economic principles [9], DHT manufacturers may significantly impact the volume of waste produced when devices reach their end of use. By designing devices with their ultimate destination in mind, or merely making the comprehensive bill of device materials available, DHT manufacturers can enable maintenance, reuse, refurbishment, and eventual recycling of their devices. Standard-setting organizations, such as CDISC, could also play a valuable role in this space, using their expertise to develop and publish standards for DHT manufacturers on reporting and sharing bills of materials.

Indeed, we know that our ontology is not comprehensive. The 500 devices we included for this analysis were selected to strike a balance between feasibility and coverage of the different types of devices we expected to find in use. In a sensitivity analysis based on the broader, keyword-based search, we found 13,502 unique trials, with a combined actual enrollment of 15,193,241 and planned enrollment of 12,615,522 participants. These numbers are obviously much larger than the ontology-driven estimates we report, but because this method is inherently over-inclusive and not device specific, it is difficult to validate these estimates without a thorough manual review—an exercise that is unscalable, beyond the scope of the present analysis, and ultimately unsuitable to support evidence-based policy-making.

However, there are instances where a specific keyword is quite similar to our ontology search. For example, one of the keywords is “freestyle libre”, which is similar to our ontology searches for the Freestyle Libre, Freestyle Libre 2, Freestyle Libre 14, and Freestyle Libre Pro DHTs. In this case, the keyword search found 185 trial records and the ontology search found a combined 65 records. The benefit of the more permissive keyword search is that it can retrieve more versions (and variations in reporting) of the Freestyle Libre devices, with the drawback of not being able to reliably conclude what specific device was involved without a manual review of the record. The benefit of the ontology search is that it permits reliable inferences about the uses of specific DHTs without the need for any manual review, because search terms tailored to maximize sensitivity and specificity for each device are saved within the ontology itself. But this, of course, comes with the drawback that trials using devices not yet in the ontology will be missed.

Thus, we fully expect our estimates of DHT use in trials to grow as more devices are added to our ontology and its sensitivity increases. To support this effort, we have made our ontology publicly available (https://bioportal.bioontology.org/ontologies/ODHT). We invite the scientific and DHT communities to contribute to improving our DHT ontology—both by inputting more devices, adding/revising information about specific devices, and suggesting improvements to the entity classification and relationship schema.

This work has important limitations: We know from our pilot work using internal company data that ClinicalTrials.gov records that involved DHTs often do not contain any evidence or mention of DHT use. This further underscore the point above that the estimates we report should be regarded as a lower-bound on the total volume of DHT use in trials. We have developed a method that supports reasonable inferences based on the data that is publicly visible in the clinical trial record, but until reporting requirements or practices change (e.g., adding fields for reporting specific DHTs within the registration record), we believe the method we have described here is laying the foundation for the best that can be done.

As we noted, phones were by far the most significant drivers of device counts based on “planned” participants. It is likely that at least some trials mentioning smart phones have “bring-your-own-device” policies, and therefore, the estimates based on “1 participant = 1 device” are over-estimating the number of devices provisioned through these trials. If these data are to inform sustainability policies, we would recommend some discounting calculation to more accurately infer the total number of devices specifically provisioned for trials. In the case of disposable smart medication, although we found only a small minority of trials using these devices today, these may become a significant source of DHT growth (and potential e-waste) in trials. At present, it is difficult to infer how many such devices are used in trials. We have based our assumptions on our experiences with such devices, but to better facilitate sustainable policies and reliable inferences about the use of these devices, we believe it would be valuable for trial registries to introduce reporting fields that would permit sponsors to report the quantity of devices.

## Conclusion

A rapidly growing number of clinical trials are employing DHTs. While some DHTs are multi-use or can be recycled, many DHTs will be “consumed” in the course of a trial and discarded. Thus, despite the great opportunities that are afforded by DHTs to clinical trial enterprise, if the industry lacks the ability to track DHT use with sufficient resolution, the result is likely to be a great deal of e-waste. We have developed a novel ontology for DHTs and a data science instrument that can provide a rich, high-resolution picture of DHT use in clinical trials. We found that thousands of trials, involving hundreds of thousands of devices, have already been completed, and many more trials (potentially involving millions more devices) are planned. But ultimately, this work represents only the beginning of what we hope will be a community-driven effort to expand the DHT ontology and more systematically report/track DHT information in trials. We believe that this effort should lead to better information across the industry, and in turn, help create a more sustainable and equitable clinical trials enterprise.

### Supplementary Information

Below is the link to the electronic supplementary material.

Supplementary file1 (XLSX 69 kb).

Supplementary file2 (XLSX 132 kb).

Supplementary file3 (XLSX 13 kb).
